# Progress in Pediatrics in 2012: choices in allergy, endocrinology, gastroenterology, hematology, infectious diseases, neurology, nutrition and respiratory tract illnesses

**DOI:** 10.1186/1824-7288-39-26

**Published:** 2013-05-08

**Authors:** Carlo Caffarelli, Francesca Santamaria, Alessandra Vottero, Sergio Bernasconi

**Affiliations:** 1 Clinica Pediatrica, Azienda Ospedaliera Universitaria, Clinical and Experimental Medicine Department, University of Parma, Parma, Italy; 2 Department of Pediatrics, Federico II University, Naples, Italy

**Keywords:** Allergy, Endocrinology, Gastroenterology, Hematology, Infectious diseases, Neurology, Nutrition, Respiratory tract illnesses

## Abstract

In this review, we summarize the progresses in allergy, endocrinology, gastroenterology, hematology, infectious diseases, neurology, nutrition and respiratory tract illnesses that have been published in The Italian Journal of Pediatrics in 2012. The induction of Treg activity by probiotics might be effective for promoting tolerance towards food allergens. Nasal cytology is useful in patients with rhinitis for diagnosing chronic non-allergic non-infectious diseases. Atopic eczema is associated both with an aberrant skin matrix and impaired systemic immune response. Therefore, isolated topical treatment may have suboptimal effect. Diagnostic work-up of exercise-induced anaphylaxis, including exercise challenge test, is necessary to reach a diagnosis. Studies may support a role for nutrition on prevention of asthma and cardiovascular diseases. Clinicians need to early identify adolescent menstrual abnormalities to minimize *sequelae*, and to promote health information. In Multiple Endocrine Neoplasia type 2B investigations include acetylcholinesterase study of rectal mucosa followed by the molecular analysis of RET mutation. Low adherence to gluten-free diet and osteopenia are common problems in children with diabetes mellitus type 1 and celiac disease. In infantile colic, laboratory tests are usually unnecessary and the treatment is based on reassurance. Prevalence of obesity and stunting is elucidated by several studies. Evidences are growing that dietetic measures are needed to prevent obesity in children with acute leukemia. Treatment studies for infectious diseases show promise for probiotics along with standard triple therapy in children with *Helicobacter pilori* infection, while zinc has no effect on pneumonia. Educational programs about the proper management of the febrile child are warranted. A new hour-specific total serum bilirubin nomogram has been shown to be able to predict newborns without hyperbilirubinemia after 48 to 72 hours of life. Newborns with hypoxic-ischemic encephalopathy present ECG and cardiac enzymes alterations leading to reduced neonatal survival. Rehabilitation programs including sensory integration therapy and motor performance, may improve activities of daily life in children with developmental coordination disorder. Aerobic exercise training in addition to chest physiotherapy might be useful in children with cystic fibrosis. Studies on effectiveness of leukotriene receptor antagonists, alone or with other drugs in preschool wheezing are needed.

## Respiratory tract illnesses

This review highlights significant 2012 The Italian Journal of Pediatrics publications. Numerous epidemiological, diagnostic and therapeutic advances in pediatrics have been reported in the last year. They provide interesting data to improve health care of children with troubling diseases.

### Allergic diseases

Current knowledge on consumption of fruits, vegetables and minerals by pregnant women and children in the first years of life and protective effects for allergies has been reviewed by Peroni DG et al. [1]. Several observational studies on antioxidants have not definitively ascertained whether antioxidants are a risk factor for asthma or whether a supplement may help in the treatment of asthma. Higher levels n-3 PUFAs (polyunsaturated fatty acids), low levels of n-6 PUFAs and high fiber intake seem to negatively correlate with the risk of wheezing. Regarding to vitamin D, both an excess and a deficiency have been associated with an increased risk of asthma. Interventional studies are needed to definitively ascertain the role of nutrients in the onset of allergic diseases. Further studies on the role of these nutrients are necessary before any conclusions can be drawn on a clinical level.

Vitaliti G et al. [2] provided a timely review on the mechanisms of cow milk allergy. Milk-specific Th2 cells have received attention for their role in IgE- mediated reactions. Th1 cells and interactions between T lymphocytes, mast cells and neurons that alter the function of the smooth muscle and the intestinal motility are involved in non-IgE mediated reactions. Treg cells are known to play a central role in tolerating foreign antigens by clonal deletion and active suppression of the immune response. The induction of Treg cellular activity by probiotics might be an intervention for promoting tolerance towards food antigens.

Allergic rhinitis is the more common allergic disease among children. Symptoms of allergic rhinitis may mimic those of other chronic diseases of the nose. Gelardi M et al. [3] provided support for understanding the role of nasal cytology in diagnosing patients with chronic non-allergic non-infectious diseases, namely non-allergic rhinitis with eosinophils (NARES), non-allergic rhinitis with mast cells (NARMA), non-allergic rhinitis with neutrophils (NARNE), and non-allergic rhinitis with eosinophils and mast cells (NARESMA). However, the therapeutic implications in differentiating subtypes of non-allergic non-infectious rhinitis remain to be elucidated. This is especially true in children since only scant data are available.

Atopic eczema is a multidisciplinary disease. Hon K-L et al. [4] reviewed 890 recent articles mainly published in pediatrics, dermatology, and allergology journal. Recent advances in pathophysiology were focused. At skin level, deficiency of ceramide and genetic defect in the filaggrin gene may result in an abnormal matrix of stratum corneum. However, authors suggested that atopic eczema should be view as a systemic disease, probably primarily driven by the immune system, to make progress in understanding the cause. They provided evidence that overactive monocytes may increase prostaglandin E and interleukin-10. Consequently, the Th2/Th1 functional balance is increased provoking elevated IL-4, 5 and 6, increased IgE levels, reduced IFN-γ production and impaired cell-mediated immune response. Therefore, isolated topical treatment may have suboptimal effect.

There is a growing level of concern regarding the potential risk of exercise-induced anaphylaxis. Povesi-Dascola C et al. [5] provided data on the role of many different foods as a prerequisite in inducing anaphylactic reactions during exertion. Furthermore, a diagnostic work-up was detailed. When it is unclear the origin of anaphylaxis, the gold standard to reach a definitive diagnosis is the exercise challenge test. No preventive drug has been shown to prevent the onset of the reaction. Therefore, avoidance of physical exercise remains the mainstay of treatment.

### Endocrinology

The most striking event in the whole process of female puberty is the onset of menstruation. Rigon F. et al. [6] provided information on the menstrual pattern and the prevalence the menstrual cycle abnormalities of Italian girls. This was a questionnaire cross-sectional study on a population-based sample of Italian adolescents aged 13–21 years attending secondary school. A total of 6,924 questionnaires were administered and 4,892 were analyzed. Adolescent girls referring persistent oligomenorrhoea, in first two years from menarche, had a higher risk for developing a persistent menstrual irregularity. They had longer bleeding periods (>6 days) and this has practical implications because it makes these adolescents potentially more susceptible to iron deficiency anemia. Clinicians need to identify menstrual abnormalities as early as possible in order to minimize their possible consequences and *sequelae*, and to promote proper health information. Health education programs for adolescents remain an important area to develop further for the purposes of prevention. In fact, adolescents should be encouraged to chart their menstrual frequency and regularity prospectively from the menarche onwards to focus their attention on the need to take care of their health relating to any menstrual problems.

Multiple Endocrine Neoplasia type 2B (MEN 2B) is an autosomal dominant complex oncologic neurocristopathy including medullary thyroid carcinoma, pheochromocytoma, gastrointestinal disorders, marphanoid face, and mucosal multiple ganglioneuromas. Medullary thyroid carcinoma is the major cause of mortality in MEN 2B syndrome, and it often appears during the first years of life. RET proto-oncogene germline activating mutations are causative for MEN 2B. The 95% of MEN 2B patients are associated with a point mutation in exon 16 (M918/T), 2%-3% of MEN 2B cases with a point mutation at codon 883. RET proto-oncogene is also involved in different neoplastic and not neoplastic neurocristopathies. Other RET mutations cause MEN 2A syndrome, familial medullary thyroid carcinoma, or Hirschsprung's disease. RET gene expression is also involved in Neuroblastoma. Early diagnosis and treatment of patients with MEN 2B are essential to their survival. The rarity of this syndrome can cause delayed diagnosis. MEN 2B is characterized by early clinical signs as nonspecific alvus disorders and, later, development of the typical facies and presence of ganglioneuromas. These signs, that precede tumor development, should suggest the diagnosis, which is based on the acetylcholinesterase study of rectal mucosa followed by the molecular analysis of RET. RET mutation detection offers the possibility to diagnose MEN 2B predisposition at a pre-clinical stage in familial cases, and to perform an early total prophylactic thyroidectomy. The surgical treatment of MEN 2B is total thyroidectomy with cervical limphadenectomy of the central compartment of the neck. When possible, this intervention should be performed with prophylactic aim before 1 year of age in patients with molecular genetic diagnosis. The protocol and diagnostic algorithm of MEN 2B that Martucciello G. et al. [7] propose seems to offer the best life expectancy to patients affected by MEN 2B syndrome. Recent advances into the mechanisms of RET proto-oncogene signaling and pathways of RET signal transduction in the development of MEN 2 and MTC will allow new treatment possibilities.

### Gastroenterology

Celiac Disease (CD) occurs in patients with Type 1 Diabetes (T1D) ranging the prevalence of 4.4-11.1% versus 0.5% of the general population. Camarca M. et al. [8] emphasized that the mechanism of association of these two diseases involves a shared genetic background: HLA genotype DR3-DQ2 and DR4-DQ8 are strongly associated with T1D, DR3-DQ2 with CD. The classical presentation of CD rarely occurs in T1D patients, but more often patients have few/mild symptoms of CD or are completely asymptomatic (silent CD). In fact diagnosis of CD is regularly performed by means of the screening in T1D patients. The effects of gluten-free diet (GFD) on the growth and T1D metabolic control in CD/T1D patient are controversial. Regarding of the GFD composition, there is a debate on the higher glycaemic index of gluten-free foods respect to gluten-containing foods; furthermore GFD could be poorer of fibers and richer of fat. The adherence to GFD by children with CD-T1D has been reported below 50%, lower respect to the 73% of CD patients, a lower compliance being more frequent among asymptomatic patients. Communication of the need of GFD in patients with T1D-CD is particularly delicate, and need a specific psychological approach. Osteopenia seems to be a new occult problem in CD patients, in T1D patients and in patients with two or three immunological diseases, depending also on GFD.

Despite infantile colic is very common in infants, origin and management are still poorly understood. Kheir AEM [9] reported that an organic cause is rare. Furthermore, there is no clear evidence that gastrointestinal, psychosocial, and neurodevelopmental disorders may play a causal role. There are some data on an association between environmental tobacco smoke and infantile colic. Laboratory tests are usually unnecessary. The treatment is based on reassurance on the benignity of the disease. Simethicone, elimination diet, oral glucose, herbal tea and calming techniques may have beneficial effects.

### Hematology

Iughetti L. et al. [10] call for measures to prevent obesity in children with acute lymphoblastic leukemia that is the most common malignancy in childhood. Continuous progress in risk-adapted treatment for childhood acute lymphoblastic leukemia has secured 8-year survival rates approaching 90%. Almost 75% of survivors, however, have a chronic health condition negatively impacting on cardiovascular morbidity and mortality. Obesity can be considered one of the most important health chronic conditions in the general population, with an increasing incidence in patients treated for childhood cancers and especially in acute lymphoblastic leukemia survivors who are, at the same time, more at risk of experiencing precocious cardiovascular and metabolic co-morbidities.

The hypothalamic-pituitary axis damage secondary to cancer therapies (cranial irradiation and chemotherapy) or to primary tumor together with lifestyle modifications and genetic factors could affect long-term outcomes. In fact, obese patients are considered at higher risk of relapse in comparison than normal weight patients, especially if they are older than 10 years of age at diagnosis. In pediatric acute myeloblastic leukemia, obese patients have greater treatment-related mortality and inferior survival compared with non-obese patients. It may be related to an inverse relationship between weight and plasma drug levels or to the effects on anticancer activity and toxicity of chemotherapy of several growth factors and lymphokines, all secreted by adipocytes.

### Infectious diseases

Fever is an extremely common sign in paediatric patients and the most common cause for a child to be conducted to the doctor’s office. The aim of the study conducted by Demir F. and Sekreter O. [11] was to identify the knowledge, attitudes and misconceptions of primary care physicians regarding fever in children. This cross-sectional study was conducted in April-May 2010 among 80 primary care physicians who work in a province with a population of 600 000 people. Physicians were surveyed using a self-administered questionnaire. The results of this study suggest that implementation of educational programs about the proper management of the febrile child are urgently needed.

Worldwide, pneumonia is the leading cause of pediatric morbidity and mortality. The reductions in the duration of severe pneumonia and its components, and overall hospitalisation might be mediated by the role of zinc in the acute phase response. Zinc supplementation might protect the lung from inflammatory states, whereas zinc deficiency might enhance airway inflammation and cellular damage. In the presence of zinc, animals had decreased inflammation of other organ systems and increased bacterial inhibition and cellular regeneration. Thus, zinc may reduce inflammation, and lower airway obstruction, in supplemented children and contribute to faster inflammation resolution time. The study conducted by Shah G.S. et al. [12] evaluates the efficacy of zinc supplementation in the treatment of severe pneumonia, beside standard anti-microbial therapy, in hospitalized Nepalese children aged 2 months to 5 years, by comparing the different outcome measures between zinc supplemented and placebo groups. Children with diagnosis of severe pneumonia were randomly assigned to receive supplementation with either oral elemental zinc or placebo. Results did not show a statistically significant reduction in duration of severe pneumonia, and duration of pneumonia as primary outcome measures. The duration of hospitalization, oxygen use and treatment failure were comparable in both groups. Thus, it appears that zinc supplementation given during acute episode has no significant effect on short-term clinical recovery from severe pneumonia in non- malnourished children. Helicobacter pylori (HP) infection is a major cause of chronic gastritis and peptic ulcers and is a risk factor for gastric malignancies, adenocarcinoma and low grade gastric mucosa associated lymphoid tissue lymphoma. In the current study Tolone S. et al. [13] evaluated whether the addition of a probiotic could improve HP eradication rates and reduce the side effects of treatment in children. The addition of probiotic formula to triple therapy (including omeprazole, amoxicillin and clarithromycin) did not increase significantly the HP eradication rates, compared to the group treated only with triple therapy. However it significantly decreased the frequency of epigastric pain, nausea, vomiting and diarrhea. In conclusion, in children with HP infection the use of probiotic formula along with standard triple therapy can be recommended as an option for decreasing overall treatment-related side effects and increasing the eradication rates.

### Neonatology

Severe neonatal hyperbilirubinemia can occur without apparent reason in term healthy infants discharged early and some of these can develop chronic bilirubin encephalopathy or kernicterus. The detection of infants without risk of severe hyperbilirubinemia has become one of the most intriguing challenges for neonatologists. The ability of physicians to recognize clinically significant jaundice and predict bilirubin levels is limited; total serum bilirubin (TSB) or Transcutaneous bilirubin (TcB) determination is often the only way to avoid such difficulty. However, TcB should be used more as a screening tool because of the stronger relationship between TSB level and the risk of hyperbilirubinemia. Romagnoli C. et al. [14] elaborated a new percentile-based hour-specific total serum bilirubin nomogram and assessed its ability to predict the absence of risk for subsequent severe hyperbilirubinaemia before discharge. In phase one 1708 healthy full term infants were studied to observe the normal trend of TSB and to elaborate the percentile-based hour specific nomogram; in phase two a prospective study was conducted in five different first level neonatal units to assess the nomogram’s predictive ability, using a single TSB value determined before discharge. They showed that the hour-specific TSB nomogram is able to predict newborn infants without significant hyperbilirubinemia only after 48 to 72 hours of life. Compared with available nomograms, the 75th percentile of this nomogram performs better.

Perinatal asphyxia is an important cause of admission to the neonatal intensive care units of newborns with multi organ dysfunction. It may progress to hypoxic-ischemic encephalopathy (HIE) and can contribute to neonatal mortality. Hypoxemia-related myocardial dysfunction in the newborns often occurs because of the effect of ischaemia on cardiac myocytes. Indeed, in these infants enzymatic markers of myocardial injury are high and appear often associated with electrocardiographic (ECG) changes. Agrawal J. et al. [15] prospectively evaluated the myocardial dysfunction in sixty neonates with HIE by clinical features, ECG and cardiac enzymes (CK Total, CK-MB and Troponin I). They concluded that significant ECG and cardiac enzymes alterations do occur in these newborn infants. Troponin I measurement may have an important role in early identification of myocardial damage secondary to ischaemia. However cardiac abnormalities are often underdiagnosed, this leading to reduced neonatal survival.

### Neurology

Developmental coordination disorders are common. Elbasan B. et al. [16] showed that problems in taking visual, tactile and proprioceptive senses and integrating them in an appropriate way leads to deficiencies in activities of daily life in children with developmental coordination disorder. Therefore, rehabilitation programs including sensory integration therapy and motor performance are necessary for increasing independence in the activities of daily life.

### Nutrition

Cardiovascular diseases (CVD) represent the leading cause of morbidity and mortality in Western countries. Guardamagna O. et al. [17] reviewed the impact of nutrition since early life on CVD. The role of prenatal nutrition is shown by inverse association between CVD mortality and birthweight, that is mainly determined by mother size and intrauterine nutrient intake. When there is a docosahexaenoic acid deficiency in the perinatal period, an increased blood pressure is observed later. Breastfeeding influences lipid metabolism, mainly determining lower levels of total cholesterol in adulthood. Other breastfeeding benefits include prevention of glucose intolerance, type 2 diabetes mellitus and development of overweight/obesity across the life course. Introduction of solid foods before 12 weeks of age is associated with higher prevalence of obese children at 7 years of age. An high-protein intake stimulates the activity of IGF-1, determining early adiposity. In newborns and young infants, an increased intake of salt could raise blood pressure. Overconsumption of energy dense complementary foods could induce excessive weight gain in infancy, with an higher risk of obesity. In childhood and adolescence, an adequate distribution between macronutrients (lipids, proteins and carbohydrates) is required for CVD prevention. Additional dietary measures include high dietary fiber intake, avoidance of sugar-sweetened beverage intake and limitation of sodium intake. A Mediterranean diet appeared to be effective in inducing clinically relevant long-term changes in CVD risk factors.

Adesina A.F. et al. [18] provided a prospective report on 960 adolescents aged 10–19 years. At variance from Western countries, the prevalence of underweight, overweight, obesity and stunting were 6.4%, 6.3%, 1.8% and 5.4% respectively. Factors which were more common in overweight adolescents were upper social class, mother with high level maternal education who spend > 3 hours a day watching television and frequent ingestion of snacks.

### Respiratory tract illnesses

Cystic fibrosis (CF) is a complex disease characterized by excessive airways mucus production, decreased exercise tolerance and breathlessness. Previous studies indicate that peripheral muscle strength, that is a physical fitness parameter, can be impaired in children with CF. Aerobic exercise training in addition to chest physiotherapy are known to improve cardiorespiratory fitness in CF. The aim of the study from Elbasan B. et al. [19] was to evaluate the effects of aerobic exercise training in addition to chest physiotherapy on physical fitness in young children with CF. The physical fitness of the 20 clinically stable patients significantly improved after 6 weeks of chest physiotherapy and aerobic exercise program. Unlike other studies, only Elbasan et al. have investigated physical fitness in patients with CF aged 5–13 years. Unfortunately, important limitations are the limited study population and the lack of a control group. Physical therapies are part of the care-management plan in CF. A consensus urgently needs to be reached on which outcome measures are appropriate for physical therapy trials. Improved therapies have progressively slowed the decline in spirometry over the past few decades, and, consequently, the sensitivity of spirometry as a measure of short- to medium-term efficacy in physical therapy trials is probably questionable. Nevertheless, spirometry remains a strong predictor of mortality, and therefore should remain a primary outcome measure in longer term trials. As current evidence suggests that physical therapy interventions are equally beneficial, treatment duration, patient preference and patient adherence may be important primary outcomes. Other outcomes, such as frequency of exacerbations, quality of life or antibiotics may also be important.

Asthma is the most common chronic disease of the airways also in young children. The review proposed by Montella S. et al. [20] summarises the most recent development on the use of the leukotriene receptor antagonists (LTRAs) in the treatment of preschool wheezing disorders. Authors highlighted the current evidences about the efficacy of montelukast (the only LTRA approved for preschool children use) in the control of wheezing, post respiratory syncytial virus (RSV) bronchiolitis and in acute asthma treatment. LTRAs are suggested for the prevention of virus-induced exacerbations, in alternative to inhaled corticosteroids (ICS) or as an add-on therapy in children who do not fully respond to ICS and to overcome the difficulties due to the use of inhalator devices or when compliance is poor. The Task Force committed by the European Respiratory Society recommends LTRAs to preschool children in both multi-trigger and episodic viral wheeze. Since current recommendations on the use of montelukast, both alone or combined with ICS, derive from evidences in older children, additional studies are needed to confirm the effectiveness of LTRAs in preschool wheezing children and to identify the subjects who may benefit from these drugs alone, or combined with other drugs. Finally, further studies should be promoted to confirm that montelukast is effective in preschool acute asthma and to investigate the LTRAs effects in post-RSV bronchiolitis.

## Competing interests

The authors declare that they have no competing interests.

## Authors’ contributions

CC conceived the study, participated in its design carried out the literature research and helped to draft the manuscript. FS participated in the design of the study, carried out the literature research and helped to draft the manuscript. AV carried out the literature research and helped to draft the manuscript. SB conceived the study, and participated in its design and coordination and helped to draft the manuscript. All authors read and approved the final manuscript.
